# Thermodynamics Affecting Glacier‐Released 4‐Nonylphenol Deposition in Alaska, USA

**DOI:** 10.1002/etc.5343

**Published:** 2022-05-17

**Authors:** Rebecca Lyons, Shaun Weatherly, Jason Waters, Jim Bentley

**Affiliations:** ^1^ Department of Chemistry, College of Arts and Sciences University of Redlands Redlands California USA

**Keywords:** Glaciers, Secondary pollutant source, 4‐Nonylphenol, Partitioning, Setschenow constant, Organic contaminants

## Abstract

Glaciers have recently been recognized as a secondary source of organic pollutants. As glacier melt rates increase, downstream ecosystems are at increasing risk of exposure to these pollutants. Nonylphenols (NPs) are well‐documented anthropogenic persistent pollutants whose environmental prevalence and ecotoxicity make them of immediate concern to the health of humans and wildlife populations. As glacier melt increases, transport of NPs to downstream environments will also increase. Snow, ice, meltwater, and till for five glaciers in the Chugach National Forest and Kenai Fjords National Park, Alaska, USA, were investigated for the presence of 4‐nonylphenol (4NP). Average concentrations for snow, ice, meltwater, and glacial till were 0.77 ± .017 µg/L snow water, 0.75 ± .006 µg/L, 0.26 ± .053 µg/L, and 0.016 ± .004 µg/g, respectively. All samples showed the presence of 4NP. Deposition of 4NP downstream from glaciers will depend more on the ionic strength of the water than organic carbon to drive partitioning and deposition. Laboratory studies of partition coefficients showed that ionic strength contributed 59% of the driving force behind partitioning, while organic carbon contributed 36%. Evidence was found for interaction between organic carbon and the aqueous phase. The 4NP Setschenow constants (*K*_s_) were determined for particle types with varying percentages of organic carbon. Values of *K*_s_ increased with the percentage of organic carbon. These relationships will shape further studies of 4NP deposition into the environment downstream of glacier outflow. *Environ Toxicol Chem* 2022;41:1623–1636. © The Authors. *Environmental Toxicology and Chemistry* published by Wiley Periodicals LLC on behalf of SETAC.

## Graphical abstract

The chemical and physical properties governing movement of 4‐nonylphenol through environmental compartments.

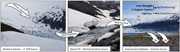

## INTRODUCTION

As global climate changes and glaciers melt at increased rates, stored persistent organic pollutants (POPs) that have been retained for decades in glacier ice and firn are increasingly being released into meltwater (Ferrario et al., 2017). Several studies have indicated that glaciers have now become secondary sources of noteworthy pollutants, including polychlorinated biphenyls, polychlorinated dibenzo‐*p*‐dioxins and dibenzofurans, and dichlorodiphenyltrichloroethane (Bogdal et al., 2010). There is additional evidence that emerging contaminants including pesticides and endocrine‐disrupting compounds (EDCs) such as 4‐nonylphenol (4NP) are present as well (Ferrario et al., 2017; Lyons et al., 2020). Both legacy and emerging contaminants are deposited in glaciers via aeolian long‐range transport mechanisms (Shevchenko et al., 2016). These are controlled by a combination of thermodynamic temperature‐driven processes, kinetically controlled advective phase‐transfer reactions, and local and regional meteorology. Because of snow scavenging and particle‐bound transport, nonpolar pollutant concentrations become amplified in snowpack to the extent that glaciers act as significant reservoirs for airborne compounds (Casal et al., 2019; X. Wang et al., 2019). As a result of amplified concentrations and faster melting, downstream ecosystems are increasingly impacted by the release of stored compounds (Owens et al., 2019; Ren et al., 2019).

While inland glaciers have been receiving recent scrutiny, maritime glaciers have been more difficult to access. However, Ademollo et al. (2021) did investigate the patterns of emerging and organic pollutants in marine water near maritime glaciers and found a positive correlation between pollutant concentrations and proximity to glaciers. Casal et al. (2017) investigated the presence of perfluoroalkyl substances (PFAS), such as perfluoroalkyl carboxylates and perfluoroalkanesulfonates (PFSAs), in the Antarctic surface snow, snowmelt, and seawater. Seawater concentrations of PFAS were shown to correlate inversely with salinity, indicating that the release of PFAS from ice and snow was the source for coastal seawater. Fresh snow concentrations of PFSAs were consistently higher than those in older snow, indicating that long‐range atmospheric transport was the primary mechanism for the movement of PFAS to Antarctica. The present study supports the idea that glacier melt in coastal regions is a potential source of pollutants into the marine environment, whether there is a local source or not. Once deposited, exposure to POPs and EDCs has well‐documented negative effects on marine wildlife, including The Authorsimmune suppression and reproductive effects (Sonne et al., 2020).

Northern Hemisphere glaciers, including Arctic glaciers, have shown a significant amount of nonpolar pollutant accumulation (Pawlak et al., 2021), and models of shifting climate patterns indicate that pollutants will likely be more prevalent over time in northern regions (X. Wang et al., 2019). For the present study, we focused on the presence and behavior of the emerging contaminant 4NP, which is the biodegradation product of the widely used surfactant nonylphenol polyethoxylate (NPE). While 4NP is not listed as a POP by the Stockholm Convention, it shares many characteristics including persistence in the environment, high octanol–water partitioning coefficients (*K*_OW_s), and toxicity (Liber et al., 1999; Lukáčová et al., 2021). It is an EDC, with significant consequences on reproductive health in humans and wildlife (Vazquez‐Duhalt et al., 2005). The estimated global production of NPE is between 45 and 230 million kg annually (US Environmental Protection Agency [USEPA], 2010). The USEPA has water quality criteria for 4NP of 6.6 μg/L for acute exposure and 1.7 μg/L for chronic exposure (USEPA 2005). Other countries have more stringent maximum contamination limits. For example, Environment Canada limits chronic exposure to 0.7 μg/L (Canadian Council of Ministers of the Environment, 2002). 4‐Nonylphenol is capable of atmospheric long‐range transport attached to particulate matter and in aerosol form. Concentrations of 4NP have been found far from anthropogenic sources in mid‐latitude glaciers (Burgess et al., 2005). This includes the Palisades and Middle Palisades glaciers of the Sierra Nevada Mountains, California, USA (Lyons et al., 2014, 2020).

The release of pollutants in glacier melt affects concentrations in downstream sediments (Pawlak et al., 2021). Preferential partitioning of nonpolar compounds to particulates with organic carbon makes sediments a potential sink (Lyons et al., 2019; Owens et al., 2019). Glacial till is generally low in organic carbon, and glacial meltwater is low in salinity (Cuffey & Paterson, 2010). Therefore, we would expect that the area least affected by 4NP release would be immediately downstream of glacier outflow. The question becomes where 4NP or other contaminants would be likely to amass.

The degree of partitioning depends on the percentage of organic carbon present (Schwarzenbach et al., 2016, pp. 275–330). The partition coefficient to particulate matter (*K*_p_) can be described as

(1) $$K_{p}=f_{\text{OC}}K_{\text{OC}}$$

where $f_{\text{OC}}$ represents the fraction of the particulate that is organic carbon and $K_{\text{OC}}$ is the partition coefficient to pure organic carbon, which can be empirically determined by finding the ratio of the equilibrium concentrations of a substance sorbed to the organic carbon solid phase and aqueous phase, shown by the equation

(2) $$K_{\text{OC}}=\frac{C_{\text{org},\text{eq}}}{C_{\text{aq},\text{eq}}}$$

where $C_{\text{org},\text{eq}}$ is the concentration of the compound sorbed to organic carbon at equilibrium and $C_{\text{aq},\text{eq}}$ is the concentration remaining in the aqueous phase.

In addition, the change in salinity that occurs when nearshore glacier meltwater mixes with coastal water may serve as a thermodynamic driving force to increase concentrations of nonpolar pollutants on particulates because the solubility of nonpolar organic pollutants is highly susceptible to the presence of ions in the solvent matrix. Huerta‐Diaz and Rodriguez (1992) demonstrated the differences in the Setschenow constant (*K*_s_) for the carbamate pesticide carbaryl (1‐naphthyl‐*N*‐methylcarbamate) in distilled water, artificial seawater, and natural seawater, with increased values in seawater. The *K*_s_ value describes the maximum concentration that can be obtained as a result of the change in salinity from deionized (DI) water and can be described by

(3) $$\mathrm{Log}\left(S_{i}^{\text{salt}}\right)-\mathrm{Log}\left(S_{i}^{\text{DI}}\right)=K_{s}C_{e}$$

where $S_{i}^{\text{DI}}$ and $S_{i}^{\text{salt}}\;$represent the solubilities in moles per kilogram of the nonelectrolyte in water and in the electrolyte solution of concentration *C*_e_, respectively. As solubility decreases, the tendency to partition to nonpolar solid phases increases (Schwarzenbach et al., 2016, pp. 275–330). The *K*_s_ value has also been used to estimate the partitioning coefficient from aqueous brine to any other phase, such as air or solid particulates. Burant et al. (2017) used the following relationship:

(4) $$\log\frac{K_{p,i}^{\text{salt}}}{K_{p,i}^{\text{DI}}}=K_{s}C_{e}$$

where $K_{p,i}^{\text{salt}}$ is the partition coefficient from the aqueous phase to the solid phase in the electrolyte solution and $K_{p,i}^{\text{DI}}$ is the partition coefficient from an aqueous deionized phase to the solid phase.

Salting‐out and partitioning to organic carbon are the two driving factors for deposition of nonpolar contaminants from glacier meltwater to sediment which will determine the extent and location of downstream distribution into sediment. Because the salting‐out coefficient had not previously been determined for 4NP, it was not known if salting‐out or partitioning would play a larger role in its eventual accumulation in sediment. Our goals were to establish the presence of 4NP in nearshore glaciers and glacial meltwater and to determine which driving force will have the greater effect on the fate of 4NP on release from glaciers. The present study investigates the presence of 4NP in nearshore glaciers of the Chugach National Forest and Kenai Peninsula, Alaska, USA, and develops the parameters for determining the eventual fate of compounds released from these glaciers. Solid–liquid and liquid–liquid extraction techniques and gas chromatography–mass spectroscopy (GC‐MS) were used to determine 4NP concentrations in various matrices.

## METHODS

### Determination of thermodynamic constants and partition coefficients

#### Materials

Laboratory testing was done on combinations of particulates and water to determine partition coefficients. Five particulate types and five water types were collected from various geographic locations to provide a range of organic carbon contents and ionic strength. The granitic soil samples were collected from the outflow of the Byron Glacier, Alaska, and Mill Creek Canyon in the San Bernardino Mountains, California, to represent low–organic carbon content particulates; “Valley Fire” soil was collected from near Forest Falls, California, to represent fire‐enriched carbon content; higher–organic carbon content soil was collected from the community garden on the University of Redlands campus; potting soil (32.5% organic carbon) and peat soil (43% organic carbon) were sourced from Lakeland Yard and Garden; and Amberlite IR‐45 resin beads were obtained from Fisher Scientific. These soil types were selected because they represent a range of environmental conditions, and the Amberlite beads were selected to provide data at the high end of the carbon content spectrum, similar to C. I. Harris and Warren (1964). Soils were collected by hand to a depth of 6 inches using a soil corer, then placed into Whirl‐Pak bags (Nasco) and stored at 4 °C until use. Nitrile gloves were worn during all soil collection.

The water types used were ultrapure reverse‐osmosis deionized water (>18 MΩ; Millipore), seawater collected from Huntington Beach, CA (33.668259 N, −118.02111 W), and surface water from Mono Lake, California (37.978411 N, −119.12499 W). The seawater was diluted with ultrapure water at a ratio of 1:1 and 1:100 to provide two additional water types, dilute seawater and freshwater, respectively. Freshwater conductivity values were compared with other natural bodies of water for consistency and found to be within 1% of freshwater ion concentrations. Water samples were collected in high‐density polyethylene (HDPE) Nalgene bottles and stored at 4 °C. Blanks were made using ultrapure water and subtracted from conductivity values. Water samples were vacuum‐filtered using Whatman cellulose filter paper (Sigma‐Aldrich). Once filtered, each water type was boiled for sterilization, placed in clean HDPE Nalgene bottles, and stored at 4 °C until use. Molar salt concentrations were determined by inductively coupled plasma spectroscopy using a Perkin‐Elmer Optima 7300 DV model. Samples were diluted 1:100 with ultrapure water and analyzed for major base cations (Ca^2+^, Mg^2+^, Na^+^, K^+^). From these values, the total ion balance and molar salt content were determined.

To determine the organic carbon content of each soil type, mass loss on combustion tests were conducted according to Nelson and Sommers (1996, pp. 1001–1006). An aliquot of each soil type was placed on a watch glass in an oven at 105 °C and left to dry for 21 h to ensure complete drying of the soil. Samples were weighed, placed in a muffle furnace at 400 °C, and then reweighed for loss on ignition.

Each water type was first brought to room temperature (20 °C). A Professional Plus Series YSI conductivity meter was used to record the temperature, conductivity, and specific conductivity of each water sample. The total dissolved solids (TDS) and ionic strength were then calculated using the following equations:

(5) TDS (mg/L)=Conductivity(µS/cm)×k

(6) $$\text{Ionic Strength}(\text{mol}/\text{kg})=\text{TDS}\times \left(2.5\times 10^{-5}\right)$$

where *k* is a modifier term which varies depending on the conductivity of the water. Values for *k* were taken from Rusydi (2018) for the various ionic strengths of water.

### Spiking the soil with 4NP

Stock solutions of 4NP (600 mg/L) were made using a pure 4NP isomeric mixture (Acros Organics) in high‐performance liquid chromatography–grade methanol (Burdick & Jackson). Particle spiking solutions consisted of a 1:100 dilution of 4NP stock in deionized water. Soils were dried in desiccators under vacuum for 24 h to ensure complete drying. They were then crushed using a mortar and pestle and sifted through a no. 35 mesh sieve (Hubbard Scientific) to homogenize particle size to 500 μm or less and prevent unwanted materials (rocks, sticks, etc.) from becoming a part of the desorption assays. Particulates were washed with hexane to remove any 4NP or other organic compounds that may have been previously sorbed to particulates. Twelve grams of each particle type were then weighed and placed in one of the 500‐ml Erlenmeyer flasks containing the spiking solutions. These were placed on a shaker table for 20 h to allow for maximum adsorption of 4NP to the particles. Solids were vacuum‐filtered from the solution using ceramic Büchner funnels and Whatman cellulose filter papers. Once filtered, each particle type was placed on a watch glass and allowed to dry in a desiccator for 1 week. Concentrations of 4NP in the particulate phase were determined by analysis of the spiking solution before and after adsorption of 4NP to the particles. In all cases, <0.05% of 4NP was left in the solution; the remainder was adsorbed to the particles.

To verify the validity of the method, a subset of each type of spiked particulate matter was set aside for analysis via direct solid–liquid desorption using USEPA Method 3550c (USEPA, 2007a). Thirty grams of particulate matter was placed in 1:1 acetone:hexane solvent. One sample was chosen for a matrix spike with 1 ml of the 200 mg/L stock solution. Samples were sonicated on an Ultrasonics sonicator (model 432B) for 3 min, then filtered. After volume reduction by Kuderna‐Danish (Organomation Associates) the filtrate was condensed using a flow of nitrogen gas to 1 ml. This was analyzed by GC‐MS to determine the initial concentration on the particulates. The mass of 4NP determined by direct desorption was within 3 ± 1.75% of the mass determined by subtraction.

### Determining partition coefficients

For each assay, 1 g of spiked particulate material was placed in a 50‐ml Erlenmeyer flask; 20.0 ml of a specific water type was added to the flask with a 10.0‐ml volumetric pipet. Each Erlenmeyer flask was then covered with aluminum foil and set aside for at least 2 weeks to allow for complete equilibration of the dosed soil particles into the water. Time to particulate:water equilibrium concentrations was determined in Lyons et al. (2019). Temperature was held constant at 10 °C. The solids were then removed from the water via vacuum filtration. The exact volume of water recovered was measured using a graduated cylinder. Liquid:liquid extraction was performed on the water with a 1:9 acetone:hexane mixture on the water according to USEPA Method 3500C (USEPA, 2007b). The extracts were then concentrated to 10 ml using a Kuderna‐Danish apparatus and further condensed to 1 ml with N_2_ gas.

Concentrations of 4NP within the condensed samples were determined using a GC‐MS equipped with a single quadrupole detector (model 5977B GC/MSD; Agilent Technologies) and a J&W HP‐5ms low‐polarity column (30 m × 250 µm × 0.25 µm; Agilent Technologies). The instrument was operated in pulsed splitless mode with an injection volume of 1 µl carried by helium gas (1.2 ml/min). The oven temperature program was as follows: an initial temperature of 45 °C (held for 3 min) was followed by a ramp from 45 °C to 300 °C (10 °C/min) before sustaining a final temperature of 300°C (held for 3 min). Injector and quadrupole temperatures were held at 250 °C and 150 °C, respectively; and ionizing electron energy was fixed at 70 eV. Standard curves were done using values of 0.25, 0.50, 1.0, 2.5, 5.0, and 10 mg/L, achieving *R* ^2^ values of 0.998 or higher. All results were above the limit of detection (0.05 mg/L). The limit of quantitation (LOQ) was 0.1711 mg/L; all reported values were at or above the LOQ. Limits of detection and LOQs were both calculated according to D. C. Harris and Lucy (2016, pp. 103–104).

Partition coefficients (*K*_p_) were calculated according to the relationship

(7) $$K_{p}=\frac{C_{\text{Part},\text{eq}}}{C_{\text{aq},eq}}$$

where $C_{\text{Part},\text{eq}}$ is the concentration of 4NP on the particulates in micrograms of 4NP per gram of soil, and $C_{\text{aq},\text{eq}}$ is the concentration in the aqueous phase in micrograms of 4NP per gram of water. It should be noted that all *K*_p_ values were determined using one concentration of 4NP. Density of the water was determined using a hydrometer (Fisherbrand; Ertco) so that water volume could be converted to mass of water (grams). After allowing the spiked particles to come to equilibrium with the water, the concentration on the particles was found by taking the initial concentration on the spiked particles and subtracting the concentration found in the water.

### Field samples

#### Site selection and sampling

**Figure 1:**
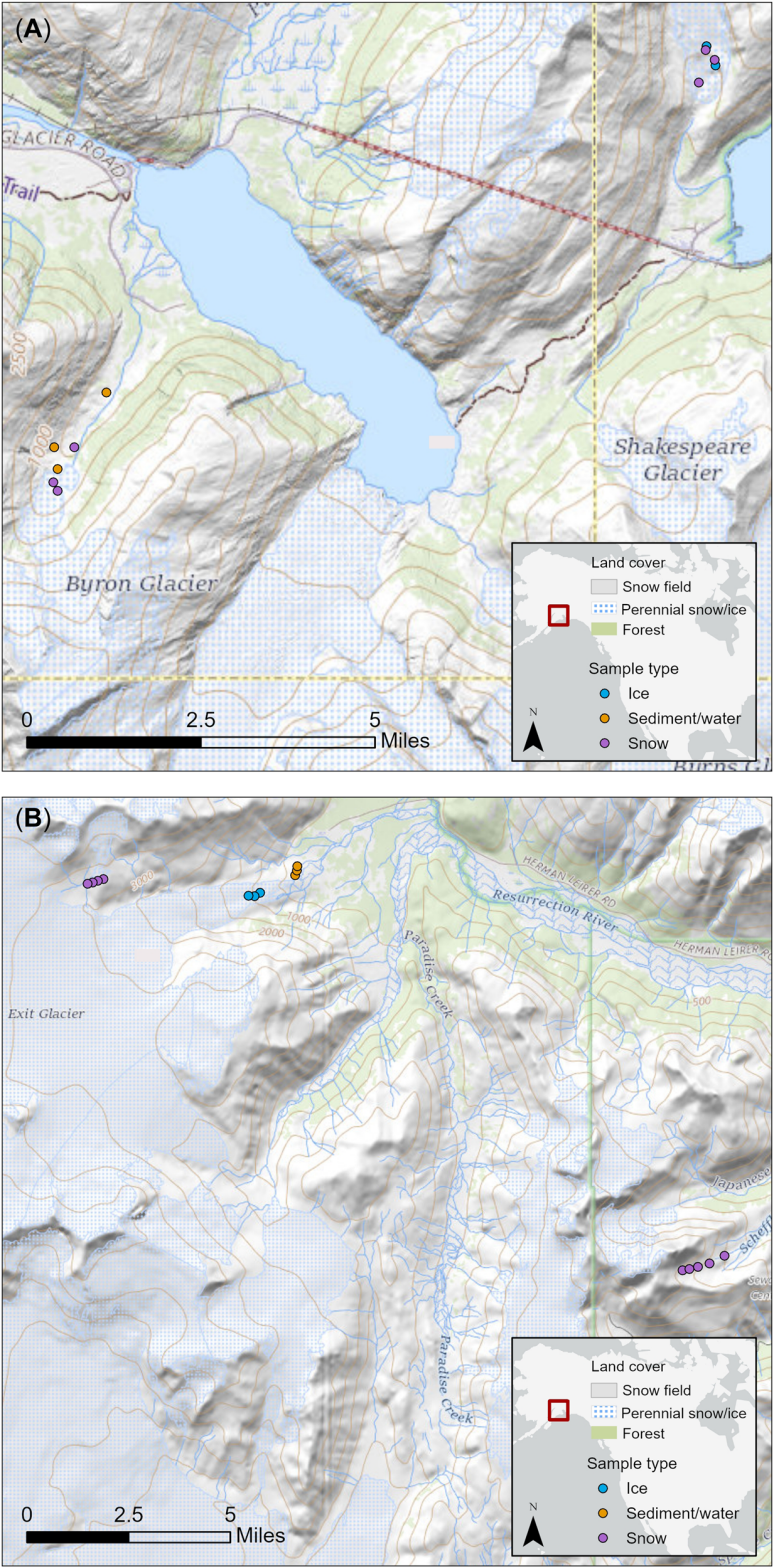
(**A**) This map depicts the northeast sampling region near the town of Whitter, Alaska, USA, showing sampling locations and types (60.745 N, −148.857 W). Sampling points on west side represent Byron Glacier samples; sampling points on northeast side are on Learnard Glacier. (**B**) This map depicts the southwest sampling region near the town of Seward, Alaska, USA, showing sampling locations and types (60.178 N, −149.651 W). Sample sites shown in the lower right‐hand corner are on Marathon Glacier.

Snow, ice, and sediment sampling took place within the Kenai Fjords National Park and Chugach State Park of the Kenai Peninsula, Alaska, from June 14 to June 21, 2021. June denotes early summer in this region, a time when seasonal snowpack has melted at lower elevations but persists among higher, glacier‐supporting elevations. All sampling locations are shown in Figure 1. Snow and ice sample sites were chosen to represent firn or seasonal snow on glaciers or permanent snow fields adjacent to the glacier of interest. Observed snowpack depth ranged from 78 to 105 cm. This was verified as seasonal snow and firn by its comparatively low density (400−500 kg/m^3^). Because sampling was done early in the summer season while there was still considerable snow cover, glacier ice was only available from two of the five glaciers (Learnard and Exit). The glaciers sampled were Byron Glacier (60.745673 N, −148.857225 W) and Learnard Glacier (60.803533 N, −148.724736 W) near Whittier, Alaska (Figure 1A) and the Harding Icefield (60.173349 N, −149.71746 W), Exit Glacier (60.178429 N, −149.651027 W), and Marathon Glacier (60.110144 N, −149.498987 W) near the town of Seward, Alaska (Figure 1B). The former two reside near the sparsely populated city of Whittier (population ~300). The latter three reside near the more populous port city of Seward (population ~2800), a center for tourism and a lucrative commercial fisheries port, according to the National Marine Fisheries Service (National Oceanographic and Atmosphere Administration, 2021).

Snow grab samples (*n* = 15) were collected using a Federal‐type snow sampler (Geoscientific). Aliquots from each core were taken from the surface, at 0.5 m, and at 1 m. For field transport, these were temporarily stored in airtight, foil‐lined Mylar® sampling bags (IMPAK). Snow was allowed to melt for 5–8 h at room temperature. An average sample volume of 600 ml snow water was obtained from each sample. This was transferred to 800‐ml stainless steel bottles (KleenKanteen) for storage and transport to Redlands, California. To prevent 4NP loss through biodegradation, bottles were refrozen and kept at <0 °C during both storage and transport. On reception at the University of Redlands, frozen samples were left to thaw so that mass of sample, conductivity (YSI Pro Plus), and total ion balance (PerkinElmer 7300DV) could be determined. Snow density was determined from the snow water mass and the snow volume measured in the field (e.g., length of snow core with known diameter). Samples were then immediately acid‐shocked to a pH of 2 (6 M H_2_SO_4_, dropwise) and filtered by means of a 0.45‐µm glass fiber filter (Whatman‐Schleicher & Schuell); 200‐ml aliquots of filtrate were then extracted in hexane (3 ×100 ml; Sigma‐Aldrich) and dried with anhydrous Na_2_SO_4_ (Sigma‐Aldrich). The extracts were concentrated down to a volume of 10 ml using a Kuderna‐Danish apparatus (Organomation Associates); then the extract volume was reduced to 1 ml under a gentle flow of nitrogen.

Glacial ice samples (*n* *=* 6) were broken free from solid ice at the ablation zone of the Learnard and Exit Glaciers using the stainless‐steel head of an ice axe and processed following the same methods as those used for snow samples. Sediment samples (*n* *=* 6) were collected using stainless steel snow shovels at sites downstream from the termini of the Exit and Byron Glaciers. Sediment samples were comprised of small rocks and coarse‐grained sediment. Rocks (>3 mm diameter) were removed; the remaining sediment was homogenized with mortar and pestle and sieved through a no. 35 sieve (Hubbard Scientific). Sediment samples were processed as the particulate samples were, according to USEPA Method 3550c (see section, *Determining partition coefficients*).

Samples of glacier meltwater downstream from the toe of the glaciers were collected in December 2021 (Figures 1); 800‐mL water grab samples were taken in stainless‐steel KleenKanteen^TM^ bottles at the same locations as the sediment samples for Exit and Byron Glaciers. On collection, the water samples were acid‐shocked to a pH of 2 and sent to Redlands, California, for analysis. Samples were extracted with previously described liquid:liquid extraction techniques, condensed, and analyzed by GC‐MS.

### Quality assurance

Snow corers, sampling bags, stainless‐steel bottles, snow shovels, glassware, and all other equipment were precleaned in deionized water, acetone, and hexane prior to use, to minimize contamination. At no point in sampling, storage, transport, or analysis did samples come in contact with products composed of synthetic polymers, which may sequester hydrophobic compounds, like 4NP, and thereby affect concentrations. Nitrile gloves were always worn during sampling and handling of equipment, to prevent contamination originating from skin care and personal hygiene products. Field and procedural blanks consisted of deionized water poured into Mylar bags on site and then analyzed alongside samples to account for possible contamination that may have occurred during sampling or processing. A subset of field blanks (*n* = 5) was spiked with 0.6 ppm 4NP and subjected to the same extraction and concentration steps as the samples. These spiked blanks yielded 92 ± 5% recovery of 4NP.

### Data analysis

Each field sample was divided into three subsamples so that laboratory analysis was performed in triplicate. Means and standard deviations of subsamples for each field sample were determined using Excel. Prediction of log *K*_p_ was carried out using linear regression models of log *K*_p_ as a function of percentage of organic carbon (POC) and ionic strength; these models were fitted using R Statistical Software's lm () function (Ver 4.1.2; R Foundation for Statistical Computing, 2021). Relative importance of POC, ionic strength, and their interaction effect in predicting log *K*_p_ were determined using the relaimpo package (Grömping, 2007).

## RESULTS

### Environmental concentration and distribution

Before the thermodynamic influences on 4NP deposition downstream of Arctic glaciers can be assessed, the extent of 4NP concentration and distribution must be determined. Four environmental media were analyzed for 4NP: glacier ice, snow and firn in permanent snow fields associated with a glacier, sediment and/or till from glacier outwash, and glacier meltwater downstream of glaciers. All environmental sample concentrations are reported in Table 1. The average snow/firn concentrations were 0.77 ± 0.017 μg/L snow water. In glacier ice, the average concentration was 0.75 ± 0.007 μg/L water. Meltwater concentrations averaged 0.525 ± 0.053 μg/L. Sediment concentrations were 0.016 ± 0.004 μg/g sediment on average. 4‐Nonylphenol has been found in glaciers in California in the United States and the Italian Alps; these regions are heavily populated and have a history of agriculture (Ferrario et al., 2017; Lyons et al., 2014). Finding 4NP in remote, coastal glaciers in the Kenai Fjords National Park and the Chugach National Forest of Alaska is noteworthy, given that the winds are primarily coming from the north/northeast (Alaska Center for Climate Assessment and Policy, 2021). While the present study does not have sufficient data for a quantitative discussion of the source of 4NP, the north/northeast wind direction suggests that Anchorage, Alaska, is a possible source. Air samples taken in other metropolitan areas show concentrations of NP of 0.165 ng/m^3^ (Ferrey et al., 2018). The present study suggests, based on the fugacity of NP, that wastewater is a potential source and accounts for the ubiquitous presence of NP in the atmosphere. According to Wania and Dugani (2003), compounds with a log octanol‐air partition coefficient (*K*_OA_) between 6.5 and 10 have the potential for long‐range transport. The log *K*_OA_ of 4NP is 7.9, making it an excellent candidate for transport and orographic deposition, as has been seen in the Sierra Nevada Mountains of California, USA (Lyons et al., 2014).

**Table 1:** The complete data set for field samples includes the elevation in meters, ionic strength for all aqueous samples, and concentration of 4‐nonylphenol

Site	Sample type	Lat	Long	Elevation (m)^a^	Ionic strength (mg/kg)	4NP (µg/L water)	SD
Byron 1	Snow	60.7521	−148.8573	752	3.6E−05	0.772	0.061
Byron 3	Snow	60.7530	−148.8582	593	2.2E−05	0.708	0.031
Byron 5	Snow	60.7565	−148.8538	409	2.2E−05	0.730	0.042
Harding 1	Snow	60.1804	−149.7072	1069	3.3E−05	0.974	0.01
Harding 2	Snow	60.2152	−149.7614	1066	2.6E−05	0.867	0.005
Harding 3	Snow	60.1989	−149.7999	1052	2.5E−05	0.777	0.0052
Harding 4	Snow	60.1746	−149.8216	989	2.3E−05	0.731	0.016
Learnard snow 1	Snow	60.7958	−148.7208	275	3.6E−05	0.787	0.02
Learnard snow 2	Snow	60.7968	−148.7227	245	3.0E−05	0.688	0.011
Learnard snow 3	Snow	60.7935	−148.7241	224	2.8E−05	0.716	0.003
Marathon 0	Snow	60.1115	−149.5007	747	3.4E−05	0.841	0.01
Marathon 1	Snow	60.1117	−149.4982	715	3.4E−05	0.752	0.0074
Marathon 2	Snow	60.1121	−149.4953	683	3.3E−05	0.727	0.0013
Marathon 3	Snow	60.1127	−149.4912	733	2.8E−05	0.717	0.012
Marathon 4	Snow	60.1141	−149.4858	608	4.5E−05	0.743	0.022
Exit 1	Ice	60.1784	−149.6509	205	1.4E−04	0.714	0.011
Exit 2	Ice	60.1778	−149.6528	214	2.1E−04	0.871	0.008
Exit 3	Ice	60.1778	−149.6550	222	3.0E−05	0.753	0.0079
Learnard ice 1	Ice	60.79524	−148.72061	275	2.3E−05	0.686	0.002
Learnard ice 2	Ice	60.7972	−148.72248	245	6.6E−05	0.726	0.001
Learnard ice 3	Ice	60.7935	−148.7241	224	7.3E−05	0.729	0.012
Byron water 1	Meltwater	60.7565	−148.8579	409	3.7E−05	0.611	0.041
Byron water 2	Meltwater	60.7621	−148.8471	399	3.6E−05	0.540	0.087
Byron water 3	Meltwater	60.7543	−148.8572	352	1.7E−04	0.873	0.112
Exit water 1	Meltwater	60.1815	−149.6383	126	9.2E−05	0.274	0.008
Exit water 2	Meltwater	60.1823	−149.6377	113	3.2E−05	0.366	0.013
Exit water 3	Meltwater	60.1831	−149.6377	116	3.6E−05	0.495	0.054
					Average % OC	Sediment (µg/g)	SD
Byron sediment 1	Sediment	60.7565	−148.8579	409	0.2406	0.0304	0.0089
Byron sediment 2	Sediment	60.7621	−148.8471	399	0.3191	0.0115	0.0094
Byron sediment 3	Sediment	60.7543	−148.8572	352	0.3403	0.0176	0.0035
Exit sediment 1	Sediment	60.1815	−149.6383	126	0.6756	0.0128	0.0017
Exit sediment 2	Sediment	60.1823	−149.6377	113	0.3002	0.0115	0.00075
Exit sediment 3	Sediment	60.1831	−149.6377	116	0.3246	0.0107	0.0011

All samples were taken and analyzed in triplicate. Standard deviations of average concentrations for field samples and analysis are pooled.

Lat = latitude; Long = longitude; 4NP = 4‐nonylphenol; SD = standard deviation; OC = organic carbon.

Summarize Elevation tool (https://pro.arcgis.com/en/pro-app/2.7/tool-reference/ready-to-use/summarize-elevation.htm).

**Figure 2:**
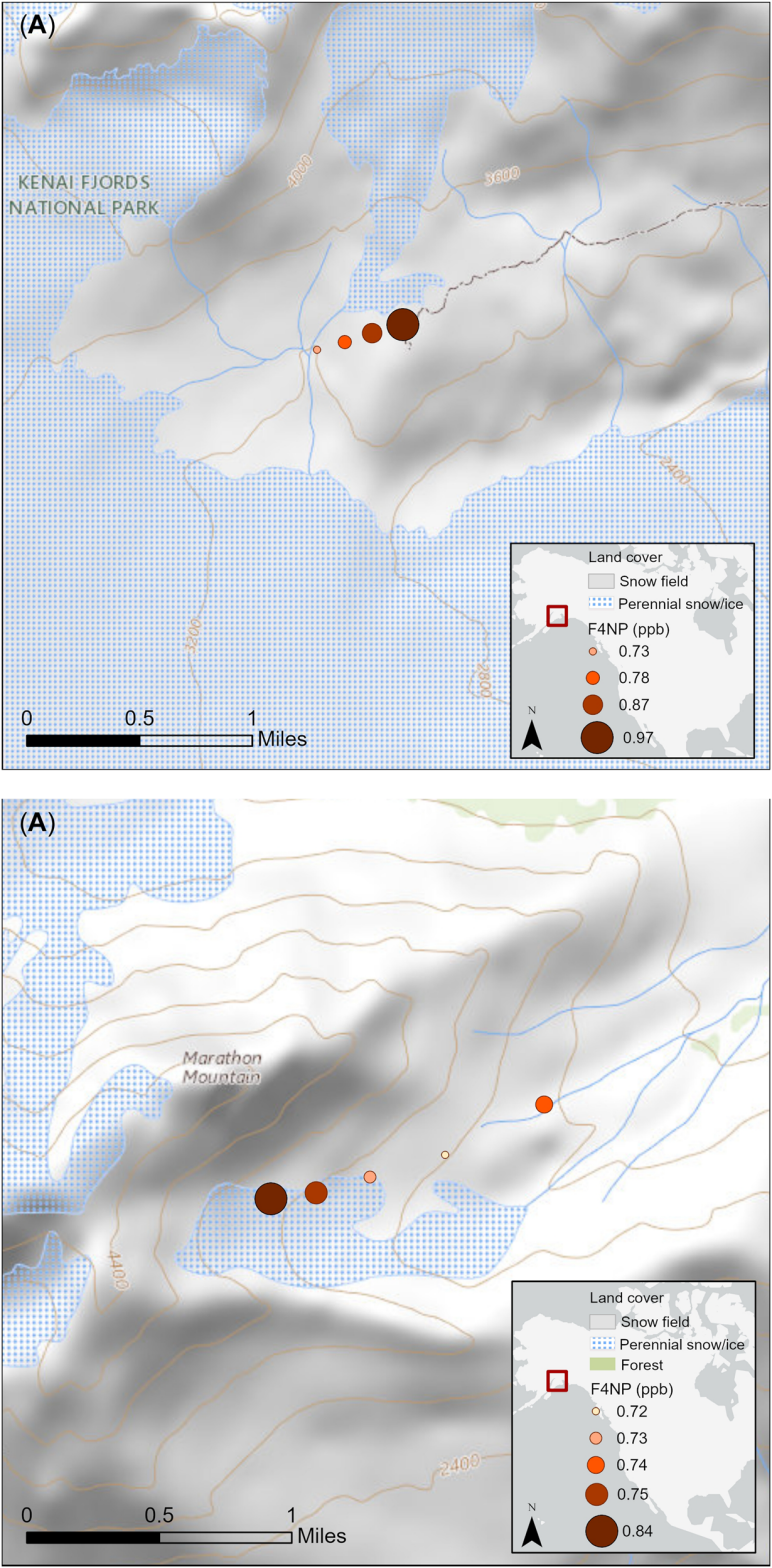
(**A**) Results from the Harding Icefield show 4‐nonylphenol concentrations increasing with elevation. Samples on the Harding Icefield were taken on the permanent snow field above Exit Glacier (60.180 N, −149.71 W). (**B**) Samples were taken on the north edge of Marathon Glacier and on the permanent snow field downslope (60.112 N, −149.50 W). Concentrations given in micrograms per liter of snow water (parts per billion). F4NP = 4‐nonylphenol.

Local distribution of 4NP on the Harding Icefield and the Marathon Glacier shows an increase in 4NP deposition with elevation (Figure 2). This is consistent with the phenomenon of orographic cold trapping of semivolatile compounds with a decrease in temperature and an increase in elevation (Daly & Wania, 2005). Semivolatile compounds will remain in the vapor phase in the warmer valley temperatures but will be scavenged at the lower temperature at higher altitudes. Snow and particle scavenging has been shown to be another important factor in the accumulation and deposition of semivolatile compounds at elevation (Franz & Eisenreich, 1998; Wania et al., 1999). Because compounds with 4NP's physical and chemical characteristics will preferentially partition to particulates, they will also accumulate through dry deposition onto glacier surfaces. Larger particles will deposit close to sources, but smaller particles will undergo long‐range transport and be subject to scavenging at higher elevations (Wagstrom & Pandis, 2011).

Average conductivity for all glacier snow and ice as water was 3.63 ± 2.86 µS/cm. Major sea spray ions are generally water‐soluble and therefore mobile within snowpack. Freeze–thaw cycles and rain on snow events mobilize soluble ions leaving surface snow and firn with relatively low conductivity (Spolaor et al., 2021). In all cases, the conductivity of the glacier snow and ice is low enough to have a minimal effect on the solubility of nonpolar organic compounds like 4NP.

For the two glaciers where sediment was collected (Byron and Exit), the outflow till was a mixture of gravel and decomposed gravel. Samples were homogenized and sieved so that the maximum particle size was 0.5 mm. Sediment collected from glacier outwash contained very little organic carbon, averaging 0.367 ± 0.155%. This relatively low POC is to be expected given that glacial till is primarily comprised of decomposed native rock and has had little opportunity to develop soil (Cuffey & Paterson, 2010). The concentrations of 4NP were correspondingly low, with an average of 0.0157 ± 0.00817 µg/g sediment dry weight. In comparison to these values, 4NP concentrations in the sediment of the Kaohsiung Harbor, Taiwan, averaged 0.101 ± 3.58 mg/g sediment dry weight (Dong et al., 2015). This value represents a high‐end example of 4NP concentration in sediment (Chen et al., 2014) because Kaohsiung Harbor has a much higher organic carbon content (1%–7%) and saline water and is near a major metropolitan area.

Meltwater was collected at approximately the same location as the sediment samples. The average concentration downstream of Exit Glacier was 0.378 ± 0.025 μg/L. Glacier ice concentrations were approximately twice this amount. This decrease in concentration was not observed in the Byron Glacier meltwater. Outflow from the Byron Glacier had an average concentration of 0.675 ± 0.08 μg/L, whereas the snow/firn samples had an average concentration of 0.736 ± 0.044 μg/L. Given the error, the concentrations in glacier snow and meltwater are essentially the same. Studies by Bizzotto et al. (2009) show that seasonal trending of pollutant release from glaciers peaks during the high‐snowmelt months (May–June). Partition coefficients determine the timing of contaminant release during snowmelt, with nonpolar compounds attached to particles being released toward the end of the melt period (Meyer et al., 2009; Meyer & Wania, 2011). Because these meltwater samples were taken in December during colder temperatures, we would expect them to represent a minimum concentration of 4NP.

### Thermodynamic driving forces

#### The effects of ionic strength and POC on partitioning

Laboratory studies were performed on combinations of particulates with different POC and water with various ionic strengths to observe their relative effects on partition coefficients. A summary of log *K*_p_ values is given in Table 2. Values for seawater molar salt concentrations were comparable to studies done on regional salinity for the Pacific Ocean (Voutchkov, 2010); for Mono Lake molar salt concentrations, values were compared with work done by He (2021) and found to be comparable.

**Table 2:** Water and soil combinations and the resulting log partition coefficient (*K*_p_) where $K_{p}=\frac{C_{\text{Part},\text{eq}}}{C_{\text{aq},\text{eq}}}$

Water type	Ionic strength^a^ (mol/kg)	Molar salt concentration (M)^b^	0.32% OC^c^	4.9% OC	12.5% OC	32.5% OC	43% OC	100% OC
Ultrapure	1.38 × 10^–5^	0	4.43 (±0.120)	4.580 (±0.210)	4.789 (±0.212)	4.811 (±0.216)	4.884 (±0.119)	5.012 (±0.158)
Freshwater	0.0060	0.1957	4.66 (±0.250)	4.74 (±0.277)	4.855 (±0.213)	4.79 (±0.1160)	5.026 (±0.077)	5.03 (±0.079)
Dilute seawater	0.421	0.978	4.71 (±0.23)	4.810 (±0.157)	5.044 (±0.162)	5.056 (±0.098)	5.065 (±0.167)	5.216 (±0.008)
Seawater	0.842	1.957	4.79 (±0.15)	4.885 (±0.252)	5.129 (±0.111)	5.17 (±0.133)	5.263 (±0.208)	5.577 (±0.173)
Mono Lake water	1.34	5.178	–d	5.053 (±0.115)	5.231 (±0.165)	5.472 (±0.203)	5.877 (±0.103)	6.929 (±0.106)

^a^ Ionic strength determined from conductivity measurements.

^b^ Molar salt concentrations determined from inductively coupled plasma measurements.

^c^ Values are means for triplicate analysis; parenthetical values give standard deviations.

^d^ Data not available.

OC = organic carbon.

**Figure 3:**
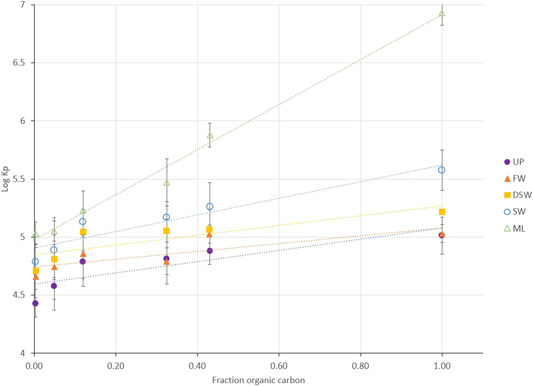
Log partition coefficient (*K*_p_) was plotted for all water types against an increase in organic carbon content in the particles. Error bars represent the first standard deviation of the log *K*_p_ for each water type. These are mean values for triplicate measurements; standard deviations are given in Table 2. UP = ultrapure; FW = freshwater; DSW = dilute seawater; SW = seawater; ML = Mono Lake water.

In Figure 3, the log *K*_p_ was plotted for all water types against POC content in the particles. The general trend for partition coefficients with increasing POC, all other conditions being held constant, shows that as the POC content increases, the partitioning to these particles increases, as was expected. An increase in organic carbon content provides more sorption sites and attraction to the particle (Schwarzenbach et al., 2016, pp. 275–330). For particles with 0.32%–43% organic carbon, linear fits can reasonably be used to compare *K*_p_ and ionic strength.

Because of the growing concern regarding marine microplastics and the potential carrying capacity that they have for organic contaminants (Ziccardi et al., 2016), the present study also includes examination of synthetic particle behavior. In the synthetic particles (Amberlite), there was almost no desorption off particles into the water under these conditions; <0.1% 4NP desorbed. This is similar to studies by Lyons et al. (2019) that saw 0.52% desorption off if 75% organic carbon particulates into deionized water. The 100% organic carbon particles appeared to behave differently from organic carbon ≤43%. When looking at the Amberlite beads (100% organic carbon), a quadratic model appears to improve fit for log *K*_p_ as a function of ionic strength, although with only five observations more work should be done to ascertain this with more certainty. In effect, Amberlite beads appear to act like an “infinite sink” for 4NP. This may be due to the synthetic nature of the particle and that both adsorption and absorption occur. As a result, this suggests that high‐carbon, synthetic particles behave differently than naturally occurring sediment across ionic strength values. These results support previous findings that microplastics represent a significant medium for environmental partitioning of hydrophobic chemicals in the ocean and may have some associated potential toxicity (F. Wang et al., 2018).

**Figure 4:**
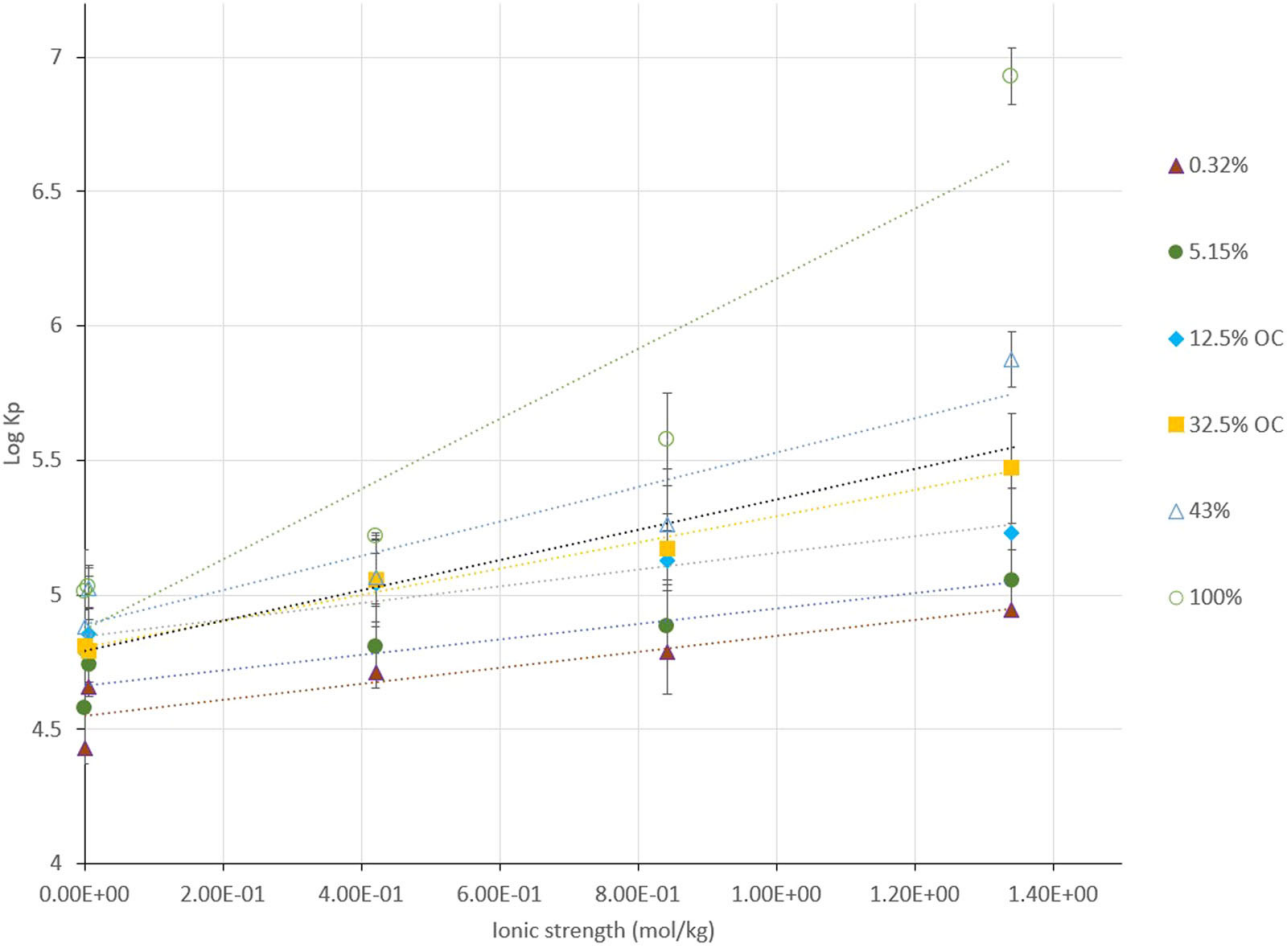
Range of ionic strengths and the resulting log of partition coefficients (*K*_p_s) are directly related. Error bars represent the first standard deviation for the log *K*_p_ for each particulate type. Values are means for triplicate measurements; standard deviations are given in Table 2. OC = organic carbon.

As ionic strength increased, partitioning to the particle increases (Figure 4), effectively showing the salting‐out of 4NP onto the solid phase. There is a substantial increase in *K*_p_ in the highest‐ionic strength water (Mono Lake water), demonstrating a nonlinear relationship at highest ionic strength relative to the other points in the model. Mono Lake has an unusual salinity, being an inland sea with water that can be up to five times more saline than ocean water. Moreover, the lake has unusual carbonate chemistry, which may also affect salting‐out (Bischoff et al., 1991).

Using a multiple linear regression model with main and interaction effects, organic carbon and ionic strength effects on log *K*_p_ can be investigated. When only environmentally relevant organic carbon percentages are modeled (≤43% organic carbon), coefficients of the linear regression model show that the interaction term between organic carbon and ionic strength is positive (ionic strength × POC). Equation 8 provides predicted log K_p_ values for combinations of ionic strength and POC. Table 3 gives the 95% confidence interval for each coefficient.

(8) $$\mathrm{Log}K_{p}=4.63+0.265\left(\text{ionic strength}\right)+6.31x10^{-3}\left(\text{POC}\right)+7.76x10^{-3}(\text{ionic strength }x\text{POC})$$

**Table 3:** Relative contribution of ionic strength, percentage of organic carbon, and their interaction to log partition coefficient for particles with 0.32%–43% organic carbon

Metric	Coefficients (Equation 8)	95% Confidence interval	*R* ^2^ contribution^a^ (not normalized)	95% Confidence interval	*R* ^2^ contribution^b^ (normalized to 100%)
Intercept	4.63	(4.53–4.74)	–	–	–
Ionic strength	0.265	(0.1135–0.4037)	0.528	(0.378–0.683)	0.590
POC	$6.31\times 10^{-3}$	(0.0024–0.0102)	0.325	(0.183–0.471)	0.363
IS:POC interaction term	$7.75\times 10^{-3}$	(0.0014–0.0154)	0.041	(0.0003–0.106)	0.047
Totals	–	–	0.894	–	1.00

^a^ Relative contribution of variables affecting log partition coefficient based on an *R* ^2^ value of 0.894 from the lm () summary and relaimpo output (R4.1.2).

^b^ Relative contributions normalized to 100%.

POC = percentage of organic carbon; IS = ionic strength.

The positive interaction term suggests that there is modification of the organic carbon sorptive capacity through contact with aqueous ions. This idea is explored further in the next section.

While Equation 8 does provide a predictive framework for log *K*_p_ values, it does not comment on the relative contribution of ionic strength versus POC. To assess the relative contribution of correlated predictor variables, the contributions of the individual effects to the overall model *R* ^2^ (0.894) can be scaled to represent a percentage of the overall *R* ^2^ value. The results of a relative importance analysis are shown in Table 3. Although ionic strength plays a larger role (59%) than organic carbon (36.3%) in driving partitioning to particulates, the difference is not significant because the confidence intervals overlap. The remainder of the log *K*_p_ variability is explained by the interaction term (4.7%), which is statistically significant.

### Determining the *K*_s_

The *K*_*s*_, or salting‐out coefficient, describes the effect of inorganic ions on the partitioning of a nonpolar solute. The constant is needed to determine the maximum concentration possible for a solute using the molar salt concentration of the aqueous environment. This value is important in determining the environmental fate of nonpolar organic chemicals that are released in coastal areas. Nonpolar compounds are expected to have large *K*_s_ values because of the high energy cost to form cavities in water (Endo et al., 2012). Molar volume and solubility have been used to estimate the salting‐out effect; however, the partition coefficient has been shown to be a better estimate of *K*_s_ in two‐phase systems, such as partitioning to sediment particles from an aqueous medium (Ni & Yalkowsky, 2003). Table 4 lists the salting‐out coefficients for the range of POC particulates in the present study based on Equation 4. The *K*_s_ increases linearly as POC increases (*R* ^2^ = 0.93). While 0.32% organic carbon does not seem to follow the trend as well as the other POC, there is considerably more variability in the results, and confidence intervals overlap with other particulate types. For Amberlite particles, which showed higher partition coefficients in all waters, the salting‐out coefficient is also significantly higher at 0.378 M^–1^. This underscores that there are two forces that drive salting‐out, attraction to the stationary phase and the molecular interactions between the water and the nonpolar compound (Schwarzenbach et al., 2016, pp. 275–330). In addition, homogeneous synthetic particles are likely to behave differently under these conditions than heterogeneous natural material.

**Table 4:** Salting out coefficient (Setschenow constant) as determined by Equation 4 using molar salt concentrations from Table 3 for all percentage of organic carbon particles

POC	Setschenow constant (*K*_s_)	95% Confidence interval^a^
0.32	0.114	(0.0271–0.261)
4.9	0.0762	(0.0334–0.119)
12.5	0.0776	(0.0350–0.120)
32.5	0.128	(0.0856–0.171)
43	0.183	(0.141–0.226)
100	0.378	(0.335–0.421)

^a^ Upper and lower 95% confidence intervals shown.

POC = percentage of organic carbon.

Salting‐out coefficients also vary considerably in the literature. It has been suggested that the hydrophobicity of sediment organic matter is modified by interactions with dissolved seawater ions (Turner, 2003). As a result, the molecular surface of the sediment organic matter is variable and affects the *K*_s_. Similarly, Panagopolous et al. (2016) determined a range of salting‐out coefficients for nonpolar compounds that were dependent on whether the compound was partitioning to organic carbon or dissolved organic carbon (DOC; Table 5). Partitioning to DOC gave higher *K*_s_ values than partitioning to solid organic carbon. This cannot be explained by a change in the driving force of the aqueous phase ionic strength alone; this demonstrates the dependence on the solid phase. Jonker and Muijs (2010) found a higher salting‐out coefficient for anthracene than previously reported in the literature by salting‐out onto a polydimethylsiloxane solid phase rather than determining *K*_s_ by solubility (Xie et al., 1997). This demonstrates that synthetic, nonpolar materials will contribute more to salting‐out coefficients than lower POC particles. All of these previous studies suggest that ionic strength and the solid phase organic carbon content affect partitioning and that interaction between the two seems likely.

**Table 5:** Comparison of Setschenow constants for compounds similar to 4‐nonylphenol in various solvents from the literature

Compound	Setschenow constant (observed)	Setschenow constant (calculated)^a^	Log *K*_OW_ b	Method used	Reference
α‐Hexachloro‐cyclohexane	0.45	0.266	3.8	Partitioning to organic carbon	Panagopolous et al. (2016)
α‐Hexachloro‐cyclohexane	1.33	0.266	3.8	Partitioning to dissolved organic carbon	Panagopolous et al. (2016)
Anthracene	0.346	0.302	4.7	Partitioning to polydimethylsiloxane	Jonker & Muijs (2010)
Anthracene	0.326	0.302	4.7	Solubility	Xie et al. (1997)
4‐Nonylphenol	0.128	0.293	4.48	Partitioning to 32.5% sediment	Present study
4‐Nonylphenol	0.378	0.293	4.48	Partitioning to Amberlite	Present study

^a^ Ni and Yalkowsky (2003).

^b^ Log *K*_OW_ values from International Programme on Chemical Safety and InChem (2014).

*K*_OW_ = octanol–water partition coefficient.

Using the *K*_OW_ linear free energy relationship that was explored in the Ni and Yalkowsky (2003) study, the *K*_s_ value for 4NP would be 0.293 using the relationship

(9) $$K_{s}=0.04\log K_{\text{OW}}+0.114$$

where the log *K*_OW_ for 4NP is 4.48 (International Programme on Chemical Safety & InChem, 2014). For 32.5% organic carbon particulates, the calculated value is nearly two times as high as the empirical salting‐out coefficient. The empirical value for salting‐out onto Amberlite is 25% lower than the calculated value. In most cases, calculated values underrepresent the empirically derived salting‐out coefficient (Table 5). This suggests that values determined with the linear free energy relationship based on *K*_OW_ consider contribution from the aqueous interactions but not necessarily dependence on partitioning to the solid phase or the interaction between organic material and the ions in solution. Therefore, to accurately determine the environmental fate of a nonpolar compound, the ionic strength, the solid phase, and any interactions should be considered. This has not been done explicitly in other studies and needs to be included for more accurate prediction of both *K*_p_ and *K*_s_.

## DISCUSSION

Once it was established that 4NP is present in all environmental compartments of northern, coastal glaciers (snow/firn, ice, till, and meltwater), the eventual downstream deposition and collection of the compound can be investigated based on the physical and chemical characteristics of the environment. While the aqueous concentrations detected are lower than the USEPA criteria for water quality of 1.7 μg/L for chronic exposure, the average aqueous concentration found in snow, ice, and meltwater (0.71 ± 0.022 μg/L) exceeds the Canadian Council of Ministers of the Environment limit of 0.7 μg/L. It is noteworthy that there were no nondetects for any of the media sampled. The amount of 4NP released from Alaskan glaciers is likely to increase as global temperatures and glacier melt rates increase. Wu et al. (2017) modeled current‐use pesticide release from glaciers in New Zealand. Release of semivolatile compounds similar to 4NP increased for all pesticides in climate change scenarios because glacial meltwater runoff increased as air temperatures increased. The present study also pointed to sediment as a potential secondary source of organic contaminants in the future.

The outwash till and sediment immediately downstream of glaciers would not be considered a major sink for nonpolar compounds like 4NP. The freshwater outflow from glaciers combined with the low POC make the glacier outwash sediment a poor reservoir for 4NP. Therefore, the concentrations of 4NP detected in the Exit and Byron outwash represent a very small fraction of the total 4NP released. Thermodynamic driving forces for partitioning to sediment become much greater downstream as POC increases. Fluvial sediments can range from 6% to 27%, depending on the nature of the riparian zone (Sutherland, 1998). Given the linear relationship between POC and log *K*_p_, the data suggest that we can choose sites for sampling that are more accessible, given a known organic carbon content to analyze glacier‐released 4NP. This could be further explored in future studies.

For glaciers that lie on a coastline and feed directly into seawater, the combined forces of high organic carbon in coastal sediment and high salinity would drive 4NP onto particles immediately. The *K*_s_ for 4NP indicates that the salt content of the water needs to increase by only a small amount to create a large increase in *K*_p_ and that particulates with even a low organic carbon content can serve as sinks. In fact, seawater concentrations of 4NP near coastal glaciers in the Kongsfjorden of Svalbard, Norway, average 10 ± 6.9 ng/L and tend to decrease exponentially with distance from the glacier (Ademollo et al., 2021). This represents aqueous concentrations an order of magnitude lower than what was found in glacier meltwater in the present study. However, this does not necessarily negate risk to aquatic organisms because there is evidence that 4NP can act in conjunction with other environmental toxins to have adverse effects even at very low concentrations (Zein et al., 2015).

Concentrations of 4NP in coastal sediments are of greater concern. While the bioavailability of 4NP to pelagic organisms decreases with partitioning to particulates, benthic organisms are still affected (Madrid et al., 2020). Oligochaetes and insects in benthic communities have demonstrated bioaccumulation and show sublethal effects of 4NP (Mäenpää & Kukkonen, 2006). Because these organisms represent the bottom of the food chain, it is likely to affect other trophic levels. Early–life stage development of marine species is directly affected by EDCs such as 4NP, and recently multigenerational effects have also been documented (DeCourten et al., 2020).

## CONCLUSION

The present study lays the groundwork for further investigation of the fate of glacier‐released 4NP. It was established that nearshore glaciers in southern Alaska have 4NP in glacier ice, snow, and firn adjacent to glaciers, glacier outwash sediment/till, and glacier meltwater. Two driving forces for 4NP deposition downstream of these glaciers were investigated: partitioning to organic carbon and salting‐out by ionic strength of the solution. Not only do ionic strength and organic carbon determine partition coefficients but the interaction of aqueous ions and organic matter plays a significant role. It has been speculated that organic matter is modified by interactions with dissolved ions. Previous estimates of the salting‐out coefficient may overrepresent how much 4NP will be driven onto particulates by the action of ionic strength. One implication of this is that there is likely to be more aqueous, bioavailable 4NP in brackish or saline water than one would expect. We found that some partitioning to organic carbon occurred immediately downstream of glaciers in the glacial till, but more deposition would be expected as the fluvial organic carbon content increases. Further work should be done to trace the progression of 4NP deposition as organic carbon content increases and determine how much of this compound reaches downstream estuaries. Future work will focus on the Byron, Learnard, and Exit Glaciers' outflow and downstream sediment in southwest Alaska. In addition, more work is needed on marine glaciers that calve directly into the ocean. Increasing concentrations of 4NP in glacial meltwater are expected as temperatures increase. The present study draws attention to the factors that affect the fate of 4NP and suggests possible reservoirs for its accumulation. The Arctic ecosystem is susceptible to multiple contaminants, of which 4NP is just one.

## Conflicts of Interest

The authors declare no conflicts of interest.

## Author Contributions Statement

**Rebecca Lyons**: Conceptualization; Investigation; Writing. **Shaun Weatherly**: Conceptualization; Investigation; Writing. **Jason Waters**: Conceptualization; Investigation; Writing. **Jim Bentely**: Formal analysis; Validation.

## Data Availability

Data, associated metadata, and calculation tools are available from the corresponding author (rebecca_lyons@redlands.edu).
